# *Lutzomyia longipalpis s.l*. in Brazil and the impact of the Sao Francisco River in the speciation of this sand fly vector

**DOI:** 10.1186/1756-3305-1-16

**Published:** 2008-06-12

**Authors:** Iliano V Coutinho-Abreu, Ivan V Sonoda, Jose A Fonseca, Marcia A Melo, Valdir Q Balbino, Marcelo Ramalho-Ortigão

**Affiliations:** 1 Departamento de Genética, Centro de Ciências Biológicas, Universidade Federal de Pernambuco, Recife, Brazil; 2 Universidade Federal do Piauí, Teresina, Brazil; Universidade Federal de Campina Grande, Campina Grande, Brazil; 3 Department of Biological Sciences, University of Notre Dame, Notre Dame, IN 46556, USA; 4 Department of Biological Sciences, University of Notre Dame, Notre Dame, IN 46556, USA

## Abstract

*Lutzomyia longipalpis s.l*. (Diptera: Psychodidae) is the principal vector of *Leishmania infantum chagasi *in the Americas, and constitutes a complex of species. Various studies have suggested an incipient speciation process based on behavioral isolation driven by the chemotype of male sexual pheromones. It is well known that natural barriers, such as mountains and rivers can directly influence population divergence in several organisms, including insects. In this work we investigated the potential role played by the Sao Francisco River in eastern Brazil in defining the current distribution of *Lu. longipalpis s.l*. Our studies were based on analyses of polymorphisms of the *cytochrome b *gene (*cyt b*) sequences from *Lu. longipalpis s.l*. available in public databases, and from additional field-caught individuals. Altogether, 9 distinct populations and 89 haplotypes were represented in the analyses. *Lu. longipalpis s.l*. populations were grouped according to their distribution in regards to the 10°S parallel: north of 10°S (<10°S); and south of 10°S (>10°S). Our results suggest that although no polymorphisms were fixed, moderate genetic divergences were observed between the groups analyzed (i.e., *F*_*ST *_= 0.184; and *Nm *= 2.22), and were mostly driven by genetic drift. The population divergence time estimated between the sand fly groups was about 0.45 million years (MY), coinciding with the time of the change in the course of the Sao Francisco River, during the Mindel glaciation. Overall, the polymorphisms on the *cyt b *haplotypes and the current speciation process detected in *Lu. longipalpis s.l*. with regards to the distribution of male sexual pheromones suggest a role of the Sao Francisco River as a significant geographical barrier in this process.

## Background

*Lutzomyia longipalpis s.l*. (Diptera: Psychodidae) is the vector of *Leishmania infantum chagasi*, the causative agent of visceral leishmaniasis (VL) in the New World [1-3]. This sand fly species has a wide, though discontinuous distribution ranging from southern Mexico to northern Argentina [4]. The pattern of distribution of this sand fly is directly associated with a notable population divergence due to a reduced gene flow, allowing the appearance of sibling species [5]. In the last few years many investigators suggested the existence of at least four sibling species of *Lu. longipalpis s.l*. in South and Central Americas, based on the study of various chromosomal and molecular markers [6-11].

Recent studies also have suggested the presence of sibling species in eastern Brazil [12,13]. These studies relied on the characterization of different male sexual pheromones, the "love songs" resulted from male wing vibration during courtship, as well as microsatellite markers and speciation genes. As many as five different chemotypes of sexual pheromones were characterized from *Lu. longipalpis s.l*. in Brazil [14]. Three of the chemotypes are pheromones of the homosesquiterpene family, 3-methyl-α-himachalene (3MαH), (S)-9-methyl-germacrene-B (9MGB) and (S)-9-methyl-germacrene-B+ (9MGB+) (the latter two can be differentiated based on the amounts of 9MGB produced). Two other chemotypes are members of the diterpene family: cembrene-1 and cembrene-2. Interestingly, different chemotypes can be associated with different male copulation sounds or love songs generated by wing flapping: 3MαH is associated with the copulation songs Pulse songtype1; 9MGB is associated with either the Pulse songtype2 or the Pulse songtype3; cembrene-1 production is associated with Burstsong [15]. No copulation songs have been associated with the production of pheromones 9MGB+ and cembrene-2 [3,14,15].

**Figure 1:**
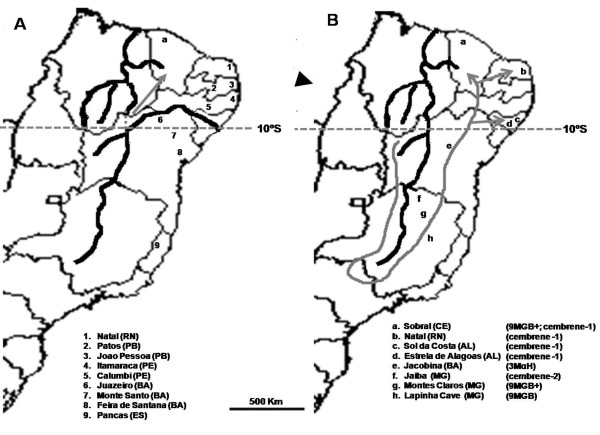
***Lu. longipalpis s.l*. across the Brazilian NE region**. Shown is the Sao Francisco River in its current course (**A**), and prior to the Pleistocene period (**B**). (**A**) *Lu. longipalpis s.l*. used in this study were sampled from various locations across the Brazilian NE (numbers 1–8) and from a single location in the SE (number 9); **a**, indicates the population in Sobral, Ceara, where two distinct male sexual pheromones (9MGB+ and cembrene-1) are found in sympatry; grey arrow indicates a secondary contact between *Lu. longipalpis s.l*. in Sobral, as suggested by [55]. According to our results we propose that this secondary contact took place following the course change of the Sao Francisco River. Gray dots (appearing in northerly direction) represent the old course of the Sao Francisco. (**B**) Original south-to-north course of the Sao Francisco River, with the distribution of the various known *Lu. longipalpis s.l*. male sexual pheromones (a-h); Arrowhead indicates expansion of *Lu. longipalpis s.l*. towards the NE region based on sub-Andean/Amazonian gene pool expansion [7]; Grey lines depicts a model for expansion of *Lu. longipalpis s.l*. populations towards SE and NE areas during the Pliocene-Pleistocene period, using the Sao Francisco River as a geographic barrier. Horizontal dashed gray lines represent the 10°S parallel.

*Lu. longipalpis s.l*. in eastern Brazil is comprised of sibling species [3]. However, studies have yet to demonstrate a link between these sibling species and geographic barriers that potentially participate in the speciation process of this sand fly. One such barrier is the Sao Francisco River, the second largest river in Brazil extending 3160 km. The Sao Francisco runs generally north behind the coastal range before turning east to form the border between the state of Bahia and the states of Pernambuco and Alagoas. It enters the Atlantic Ocean between the states of Alagoas and Sergipe (Figure 1A). Until the Mindel glaciation period (0.4 million years ago – MY) the Sao Francisco ran northward towards the State of Piaui (Figure 1B). During the Mindel glaciation, the course of the river was altered to its current location [16], as shown in Figure 1.

Natural geographical barriers such as rivers have been shown to significantly influence genetic diversification and speciation in distinct taxa [17-21]. Thus, we analyzed the population structuring of *Lu. longipalpis s.l*. in eastern Brazil based on different haplotypes of the *cytochrome b *gene (*cyt b*), and investigated the role of the Sao Francisco River in the speciation of this sand fly. Our results suggest that the natural barrier represented by the Sao Francisco River in *Lu. longipalpis s.l*. has played a significant role in the development of sibling species of this sand fly. The data are supported by evidence from recent studies on the geological history of the Sao Francisco River [16]. To our knowledge, this work is the first to link the ongoing speciation of *Lu. longipalpis s.l*. in eastern Brazil with a significant natural barrier.

## Methods

### Sand flies

*Lu. longipalpis s.l*. used in this study were captured in five separate locations (Figure 1 and Table 1) using CDC light traps. Trapping of sand flies was done as described previously [22]. Adult sand flies were killed by freezing at -20°C and identified as described [4].

**Table 1:** Haplotype distribution in *Lu. longipalpis s.l*. populations investigated in this study.

**Populations**	**Coordinates**	**N**	**Groups**	**Haplotypes**
Natal	5°77'S/35°30'W	20	5°S-10°S	***Ll-1***(2); ***Ll-2***; ***Ll-7***; ***Ll-8***(2); *Ll-9*; *Ll-10*(2); **Ll-14**; *Ll-15*(4); Ll-16; Ll-17; **Ll-18**; Ll-19; Ll-20; Ll-21
Patos^1^	7°02'S/37°29'W	3	5°S-10°S	*Ll-9; Ll-10*; Ll-37
João Pessoa^1^	7°07'S/34°86'W	2	5°S-10°S	*Ll-15*; Ll-36
Itamaracá*	7°45'S/34°49'W	12	5°S-10°S	***Ll-1*; ***Ll-3*(3); *Ll-9*(4); Ll-25; Ll-26; Ll-27
Calumbi^1^	7°59'S/38°18'W	3	5°S-10°S	***Ll-8***; Ll-34; Ll-35
Juazeiro	9°47'S/40°49'W	16	5°S-10°S	***Ll-1***; ***Ll-2***; *Ll-3*(3); Ll-4; Ll-5; ***Ll-7***(3); ***Ll-8***; *Ll-9*(2); *Ll-10*; Ll-12; Ll-13
Mte. Santo	10°44'S/39°34'W	10	>10°S	***Ll-2***(7); ***Ll-14*(1)**; Ll-32; Ll-33
F. de Santana	12°20'S/39°02'W	8	>10°S	**Ll-1**; **Ll-7**; ***Ll-14***;**Ll-18 **(2); Ll-29; Ll-30; Ll-31
Pancas	19°20'S/40°83'W	16	>10°S	***Ll-2*(4); Ll-8 **(6); Ll-22 (3); Ll-23; Ll-24 (2)

Population groups were separated according to their geographic coordinates. Haplotypes shared between population groups are shown in bold; haplotypes shared by the populations within a group is shown in italics and underlined. Populations from which haplotypes were identified in this work are marked by (^1^), and the haplotypes obtained from the population from Itamaracá is marked by (*).

### DNA Extraction, Amplification and Sequencing

The DNA samples were prepared as reported previously [22], and amplification of *cyt b *was performed using the primers N1N-5'GGCAYWTTGCCTCGAWTTCGWTATGA3' and CB3-5'CAYATTCAACCWGAATGATA3' [23]. PCR reactions were routinely carried out in 50 μl volume containing 10 pmoles of each primer, 1 ng of *Lu. longipalpis s.l*. DNA, 5 μl of 10× PCR buffer with 2 mM of MgCl_2 _(Biotools, B&M Labs, S.A. Madrid, Spain), 60 μM of dNTPs and 1.5 units of Taq DNA polymerase (Biotools). PCR reactions were performed as follows: a hot start at 94°C/3 min, followed by five cycles at 94°C/30 sec, 38°C/30 sec and 72°C/90 sec, and 35 cycles at 94°C/30 sec, 42°C/30 sec and 72°C/90 sec. The PCR products were purified with the Nucleospin Extract kit (Macherey-Nagel Inc., Easton, PA) and cycle-sequenced using the BigDye sequencing kit (Applied Biosystems, Foster City, CA), according to the manufacturer instructions. The sequencing reactions were performed using an ABI PRISM^® ^3100 sequence analyzer (Applied Biosystems). Each sample was sequenced twice and the sequencing quality was assessed using Phred [24] with a cutoff value of 25. The haplotypes were assembled using CAP3 [25].

### Analysis of Genetic Variability

Analyses of genetic variability were performed in 34 haplotypes identified from *Lu. longipalpis s.l*. used in this work, including haplotypes described previously [26,27]. The indexes of genetic variability were estimated using DnaSP 4.10 [28]. The parameters measured were: the number of haplotypes (*h*); the haplotypic diversity (*Hd*); the number of segregating sites (*S*); the pairwise nucleotide diversity (*π*); the net nucleotide substitution (*d*_*A*_); and the average number of nucleotide substitutions (*d*_*XY*_). The *θ *parameter (*2N*_*e*_*μ*) was estimated for *θπ *and *θ*_*s*_. *θπ *is based on the average pairwise number of differences between sequences [29] and *θ*_*s *_is estimated from the number of segregation sites in a population [30]. The following tests were performed to determine whether the pattern of polymorphisms were in accordance with the model of neutral evolution: *D *[31], D* and F* [32], and *Fs *[33]. The *Fs *test was also used to assess population expansion.

The extent of nucleotide differentiation (*F*_*ST*_) [34] and the level of gene flow (*Nm*) [35] among the populations also were analyzed using DnaSP 4.10 [28]. The *F*_*ST *_values were plotted in a matrix and used to create a distance-based Neighbor-Joining (NJ) tree using MEGA v3.1 [36]. The Kimura-2P (*K2P*) genetics distance between clades of the NJ tree was also calculated using the MEGA v3.1 software. The genetic distances were used to calculate the time of divergence between clades, based on the rate of mutation of 2.3% sequence divergence per million years [37]. The minimum spanning networks and the mismatch distributions were obtained using Arlequin v2.0 [38]. Raggedness tests were performed to determine whether mismatch distributions fit the model of stable or expanding populations [39-41]. Also, we assessed the significance of the effect of geographic distance on differentiation via the Mantel test [42]. This test was based on the linear regression between the value of the *F*_*ST*_/(1 - *F*_*ST*_) [43] and the geographical straight-line distance (in kilometers, Km) between each population pair. Geographic distances between capture sites were obtained from the Coordinate Distance Calculator [44]. Results of the Mantel test were a reflection of 1,000 permutations using Mantel v1.18 [45].

The populations analyzed in this study were combined into two groups, taking into account the sand fly distribution in relation to the 10°S parallel. Thus, individuals that were originally captured north of the 10°S parallel were named <10°S; and those captured south of the 10°S parallel, >10°S

## Results

In this work we analyzed phylogeographic patterns of natural populations of *Lu. longipalpis s.l*.. in the Brazilian NE region. Our studies were based on polymorphisms of a 261 bp DNA fragment from the 3' end of the *cyt b *gene. The phylogenetic signal shown by this short fragment was higher than those presented by fragments of 318 and 489 nucleotides (unpublished data). Our analyses included twenty nine haplotypes previously described [26,27] and five novel haplotypes identified from *Lu. longipalpis s.l*. from the Brazilian NE.

Out of 11 *Lu. longipalpis s.l*. individuals sequenced, nine distinct haplotypes were identified, including seven described earlier [26,27]: Ll-8 from Calumbi; Ll-3 (present in two individuals) and Ll-9 from Itamaracá; Ll-15 from João Pessoa; and Ll-9 and Ll-10 from Patos (Table 1). Five novel haplotypes were named Ll-34 and Ll-35 (from Calumbi), Ll-36 (from João Pessoa), and Ll-37 (from Patos). A total of 34 haplotypes (including the ones described by [26,27]) and their frequencies were analyzed as indicated above. The haplotypes differed up to six mutation steps and the polymorphic sites displayed no more than two variant nucleotides. The relationship between transition and transversion among the haplotypes was 25:1, with only five substitutions being non-synonymous (at position 26, a Leu to Ser change, L26S; then, S53N, P161L, V190M, N254S).

Our data shows that Ll-2 is the most frequent haplotype and the haplotypic diversity is high (0.947) despite the geographically widespread distribution of haplotypes Ll-1, Ll-2 and Ll-7 (Tables 2 and 3). Furthermore, no genetic differences were found between the populations of Juazeiro and Natal, Juazeiro and Patos, Natal and Patos, Natal and Calumbi, and Patos and Calumbi. In contrast, the highest genetic distance was estimated between populations of Itamaraca and Monte Santo (*d*_*A *_= 0.496%; Table 4).

**Table 2:** Polymorphic sites of 34 haplotypes of ten *Lu. longipalpis s.l*. populations from the Eastern Brazil.

**Haplotypes**	**Segregating sites**																										**Number of haplotypes in each population**											
									**1**	**1**	**1**	**1**	**1**	**1**	**1**	**1**	**1**	**1**	**1**	**1**	**1**	**1**	**1**	**1**	**2**	**2**			**N**	**P**	**J**	**I**	**C**	**J**		**M**	**F**	**P**

	**2**	**3**	**4**	**5**	**6**	**6**	**8**	**9**	**0**	**0**	**1**	**4**	**4**	**4**	**5**	**5**	**5**	**5**	**6**	**6**	**7**	**9**	**9**	**9**	**4**	**5**			**T**	**T**	**P**	**T**	**L**	**Z**		**S**	**S**	**N**
	**6**	**6**	**8**	**3**	**0**	**9**	**1**	**9**	**2**	**5**	**1**	**1**	**4**	**7**	**0**	**3**	**6**	**9**	**1**	**2**	**4**	**0**	**2**	**3**	**0**	**4**			**L**	**S**	**S**	**A**	**B**	**R**		**T**	**N**	**C**
**Ll-1**	T	C	T	G	T	T	G	T	A	T	T	A	G	A	T	T	A	C	C	C	C	G	A	T	A	A			2			1		1			1	4
**Ll-2**	.	.	.	.	.	.	.	.	.	.	.	G	.	.	.	.	.	.	.	.	.	.	.	.	.	.			1					1		7		
**Ll-3**	.	.	.	.	.	.	.	.	.	.	.	.	.	.	.	.	.	T	.	T	.	.	.	.	G	.						3		3				
**Ll-4**	.	.	.	.	.	.	.	.	.	.	.	G	.	.	C	A	G	T	.	T	.	.	.	.	.	.								1				
**Ll-5**	.	.	.	.	.	.	A	C	.	C	.	.	A	.	.	.	.	.	.	T	T	.	.	.	.	.								1				
**Ll-7**	.	.	.	.	.	.	.	.	.	.	.	.	A	.	.	.	.	.	.	.	.	.	.	.	.	.			1					3			1	6
**Ll-8**	.	.	.	.	.	.	A	.	.	.	.	G	.	.	.	.	.	.	.	.	.	.	.	.	.	.			2				1	1				
**Ll-9**	.	.	.	.	.	.	A	.	.	.	.	.	.	.	.	.	.	.	.	.	.	.	.	.	.	.			1	1		4		2				
**Ll-10**	.	.	.	.	.	.	A	.	.	.	.	G	.	.	.	.	.	T	.	T	.	.	.	.	.	.			2	1				1				
**Ll-12**	.	.	.	.	.	.	.	.	G	.	.	G	.	.	.	.	G	T	.	T	.	.	.	.	.	.								1				
**Ll-13**	.	.	.	.	C	.	.	.	.	.	.	.	.	.	.	.	.	.	.	T	.	.	.	.	.	.								1				
**Ll-14**	.	.	.	.	.	.	.	.	.	.	.	G	A	.	.	.	.	.	.	.	.	.	.	.	.	.			1							1	1	
**Ll-15**	.	.	.	.	.	.	.	.	.	.	.	.	.	.	.	.	.	.	.	T	.	.	.	.	.	.			4		1							
**Ll-16**	.	.	.	.	.	.	A	.	.	.	.	G	.	.	.	.	.	T	.	T	T	.	.	.	.	.			1									
**Ll-17**	.	.	.	.	.	.	.	.	.	.	.	G	.	.	.	.	.	T	.	T	.	.	.	.	.	.			1									
**Ll-18**	.	.	.	.	.	.	A	.	.	.	.	G	A	.	.	.	.	.	.	.	.	.	.	.	.	.			1								2	
**Ll-19**	.	.	.	.	.	.	A	.	.	.	.	G	A	.	.	.	.	T	.	T	T	.	.	.	.	.			1									
**Ll-20**	.	.	.	.	.	.	.	.	.	.	.	.	.	.	.	.	.	T	.	T	T	.	.	.	.	.			1									
**Ll-21**	.	.	.	.	.	.	.	.	.	.	.	.	A	.	.	.	.	.	.	.	T	.	.	.	.	.			1									
**Ll-22**	.	.	.	A	.	.	.	.	.	.	.	G	.	.	.	.	.	T	.	.	.	.	.	.	.	.												3
**Ll-23**	.	.	.	.	.	.	.	.	.	.	.	G	.	.	.	.	.	T	.	.	T	.	.	.	.	.												1
**Ll-24**	.	.	.	.	.	.	.	.	.	.	.	G	.	.	.	.	.	T	.	.	.	.	.	.	.	.												2
**Ll-25**	.	.	.	.	.	.	.	.	.	.	.	.	.	.	.	.	.	T	.	T	.	.	.	.	.	.						1						
**Ll-26**	.	.	.	.	.	.	.	.	.	C	.	.	.	.	.	.	.	.	.	T	.	.	.	.	G	.						1						
**Ll-27**	.	.	.	.	.	.	.	.	.	.	.	.	.	.	.	.	.	.	T	.	.	.	.	.	.	.						1						
**Ll-29**	.	.	.	.	.	.	.	.	.	.	.	G	.	G	.	.	.	.	.	.	.	.	.	.	.	.											1	
**Ll-30**	.	.	C	.	.	.	.	.	.	.	.	G	A	.	.	.	.	.	.	.	.	.	.	.	.	.											1	
**Ll-31**	.	.	.	.	.	.	A	.	.	.	.	G	.	.	.	.	.	.	.	T	.	.	.	C	.	.											1	
**Ll-32**	.	.	.	.	.	.	A	.	.	.	.	G	.	.	.	.	.	.	.	T	.	A	.	C	.	.										1		
**Ll-33**	.	.	.	.	.	.	A	.	.	.	.	G	.	.	.	.	.	.	.	.	.	.	G	.	.	.										1		
**Ll-34**	.	.	.	.	.	.	A	.	.	.	.	.	A	.	.	.	.	.	.	.	T	.	.	.	.	.							1					
**Ll-35**	.	.	.	.	.	.	A	.	.	.	.	G	.	.	.	.	G	T	.	T	.	.	.	.	.	.							1					
**Ll-36**	.	T	.	.	.	C	.	.	.	.	C	.	.	.	.	.	.	.	.	.	.	.	.	.	.	.					1							
**Ll-37**	.	.	.	.	.	.	.	.	.	.	.	G	.	.	.	.	.	T	.	T	T	.	.	.	.	.				1								

*Lu. longipalpis s.l*. populations are indicated by a vertival 3 letter code: Natal (NTL), Patos (PTS), João Pessoa (JPS), Itamaracá (ITA), Calumbi (CLB), Juazeiro (JZR), Monte Santo (MST), Feira de Santana (FST) and Pancas (PNC). GenBank accession numbers for haplotypes Ll-1 to Ll-33 were reported in [26, 27]. GenBank accession numbers for Ll-34 to Ll-37 are EF107662 to EF107665, respectively.

**Table 3:** Parameters of genetic diversity for *Lu. longipalpis s.l*. populations used in this study.

Groups	***n***	***h***	***S***	***Hd***	***π***	***θs***	***θπ***
<10°S	55	26	18	0.9475 (0.014)	0.01218 (0.00086)	0.01507 (0.0053)	3.934
>10°S	34	14	12	0.8645 (0.044)	0.00710 (0.00097)	0.01124 (0.0045)	2.935
Total	89	33	24	0.9460 (0.011)	0.01128 (0.0007)	0.01817 (0.00572)	4.743

Standard deviations are in parenthesis.

**Table 4:** The number of migrants (*Nm*, italics), pair wise nucleotide diversity (*π*, bold numbers) and net nucleotide substitution per site (*d*_*A*_) between ten population of *Lu. longipalpis s.l*.

	NTL	PTS	JPS	ITA	CLB	JZR	MST	FSN	PNC
NTL	**0.00974**	-	*6.41*	*3.79*	-	-	*2.33*	*5.50*	*2.18*
PTS	-0.034	**0.01277**	*2.75*	*5.41*	-	-	*1.88*	*2.19*	*5.13*
JPS	0.069	0.255	**0.01533**	*29.50*	*2.00*	*17.96*	*1.45*	*1.86*	*1.07*
ITA	0.138	0.102	0.021	**0.00933**	*2.82*	*102.62*	*0.73*	*1.10*	*0.78*
CLB	-0.021	-0.255	0.383	0.218	**0.01533**	*77.83*	*2.74*	*19.50*	*5.84*
JZR	-0.005	-0.038	0.042	0.006	0.010	**0.0145**	*1.76*	*3.36*	*1.78*
MST	0.135	0.238	0.353	0.496	0.187	0.280	**0.00519**	*4.89*	*4.76*
FSN	0.073	0.255	0.335	0.430	0.032	0.179	0.076	**0.00958**	*2.00*
PNC	0.174	0.089	0.489	0.475	0.089	0.282	0.00056	0.189	**0.00556**

*Lu. longipalpis s.l*. populations are: Natal (NTL), Patos (PTS), João Pessoa (JPS), Itamaracá (ITA), Calumbi (CLB), Juazeiro (JZR), Monte Santo (MST), Feira de Santana (FSN) and Pancas (PNC).

**Table 5:** Estimated fixation index (*F*_*ST*_) and geographic distances (in Km) between ten *Lu. longipalpis s.l *populations.

	NTL	PTS	JPS	ITA	CLB	JZR	MST	FSN	PNC
NTL	-	**234.46**	**92.45**	**178.91**	**361.94**	**702.29**	**665.28**	**773.50**	**1578.10**
PTS	*0.0000*	-	**226.67**	**304.88**	**138.43**	**476.70**	**468.99**	**610.38**	**1424.70**
JPS	*0.0779*	*0.1538*	-	**97.67**	**330.56**	**662.0**	**605.46**	**699.18**	**1496.96**
ITA	*0.1131*	*0.0846*	*0.0167*	-	**385.00**	**697.38**	**617.45**	**685.97**	**1464.79**
CLB	*0.0000*	*0.0000*	*0.2000*	*0.1504*	-	**340.96**	**334.47**	**487.81**	**1299.15**
JZR	*0.0000*	*0.0000*	*0.0271*	*0.0048*	*0.0064*	-	**172.61**	**342.82**	**1058.43**
MST	*0.1954*	*0.2097*	*0.2562*	*0.4058*	*0.1544*	*0.2214*	-	**186.31**	**971.40**
FSN	*0.1019*	*0.1860*	*0.2121*	*0.3127*	*0.0250*	*0.1296*	*0.0928*	-	**814.43**
PNC	*0.1959*	*0.0889*	*0.3188*	*0.3893*	*0.0789*	*0.2193*	*0.0950*	*0.2000*	-

*Lu. longipalpis s.l*. are as shown on Table 4. *F*_*ST *_(italics) and distances in Km (bold) are shown

**Figure 2:**
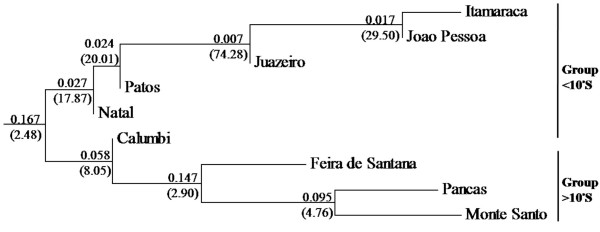
**F_ST _distance-based Neighbor-Joining tree**. Group <10°S is represented by the six populations: Calumbi, Natal, Patos, Juazeiro, João Pessoa and Itamaracá. Group >10°S encompasses the populations from Pancas, Monte Santo and Feira de Santana. Values of *F*_*ST *_and *Nm *(in parenthesis) for branches separating two populations are provided.

A *F*_*ST *_distance-based Neighbor-Joining (NJ) tree was obtained for all populations investigated in this study. Two clades were identified: (I) including the populations of Natal, Patos, Juazeiro, João Pessoa, and Itamaracá; and (II) including the populations of Calumbi, Pancas, Monte Santo, and Feira de Santana (Figure 2). In light of the low level of genetic structuring and geographic distribution the two clusters seen in Figure 2 were referred to as group <10°S (for populations north of 10°S parallel) and group >10°S (for populations south of 10°S parallel). According to its geographic localization the population of Calumbi was also included in <10°S.

The net nucleotide substitution (*d*_*A*_) between the groups <10°S and >10°S was 0.217% (Table 6). The estimates of haplotypic diversity within each group were high (Table 3), although some haplotypes (Ll-1, Ll-2, Ll-7 e Ll-8) presented a widespread geographic distribution. The average number of nucleotide differences (*π*) varied from 0.7% to 1.2% per site, or from 1.85 to 3.18 per nucleotide (Table 3), and no site were fixed at different nucleotides between groups. Within the 55 haplotypes from the group <10°S, 18 (32.7%) were singletons. In contrast, a lower proportion of singletons (20.6%, or 7 out of 34) was observed within the group >10°S.

**Table 6:** Genetic differences between population groups.

Groups	<10°S	>10°S
<10°S	*0.078 (5.92)*	0.217 (1.2) *
>10°S	**0.184 (2.22)**	*0.136 (3.16)*

Estimated fixation index (*F*_*ST*_) and the number of migrants (*Nm*, in parenthesis) are shown in bold. The percentage of genetic distances *d*_*A *_and *d*_*XY *_(in parenthesis) between groups <10°S and >10°S are marked (*). The *F*_*ST *_index of each group is shown in italics (diagonal).

**Figure 3:**
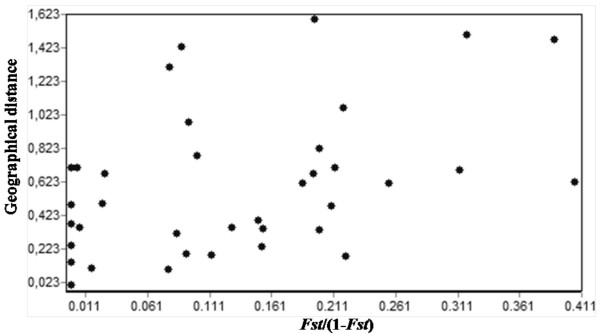
**Minimum Spanning Networks (MSN)**. Haplotypes are coded according to the population group within which they are found. Black circles represent shared haplotypes and colored circles represent haplotypes unique to a population within a group. Size of circle is proportional to the haplotypic frequency, and each cross bar indicates one nucleotide substitution between haplotypes. Dashed lines indicate alternative branching between haplotypes. (**A**) MSN obtained from all 34 haplotypes from *Lu. longipalpis s.l*. used in this study. The black circles are haplotypes shared by all population groups. Colored code: group <10°S (green); and group >10°S (red). (**B**) MSN for group <10°S: Black circles are haplotypes shared by populations within this group. Colored circles are haplotypes from Juazeiro (green), Natal (light blue), Itamaracá (grey), João Pessoa (orange), Patos (white) and Calumbi (dark blue). (**C**) MSN for group >10°S: Black circles are haplotypes shared by populations within this group. Colored circles are haplotypes specific to Pancas (yellow), Monte Santo (grey) and Feira de Santana (pink).

Intra-population group analysis showed values of genetic structuring (*F*_*ST*_) and gene flow (*Nm*) of 0.078 and 5.92 for the group <10°S (Table 6). Equivalent results were also obtained for the group >10°S (*F*_*ST *_= 0.136 and *Nm *= 3.16, Table 6). The values of *F*_*ST *_among the two groups support moderate genetic divergence and higher gene flow values (*F*_*ST *_= 0.184 and *Nm *= 2.22, Table 6), whereas the pattern of genetic structuring did not follow the model of isolation by distance (Figure 3).

**Figure 4:**
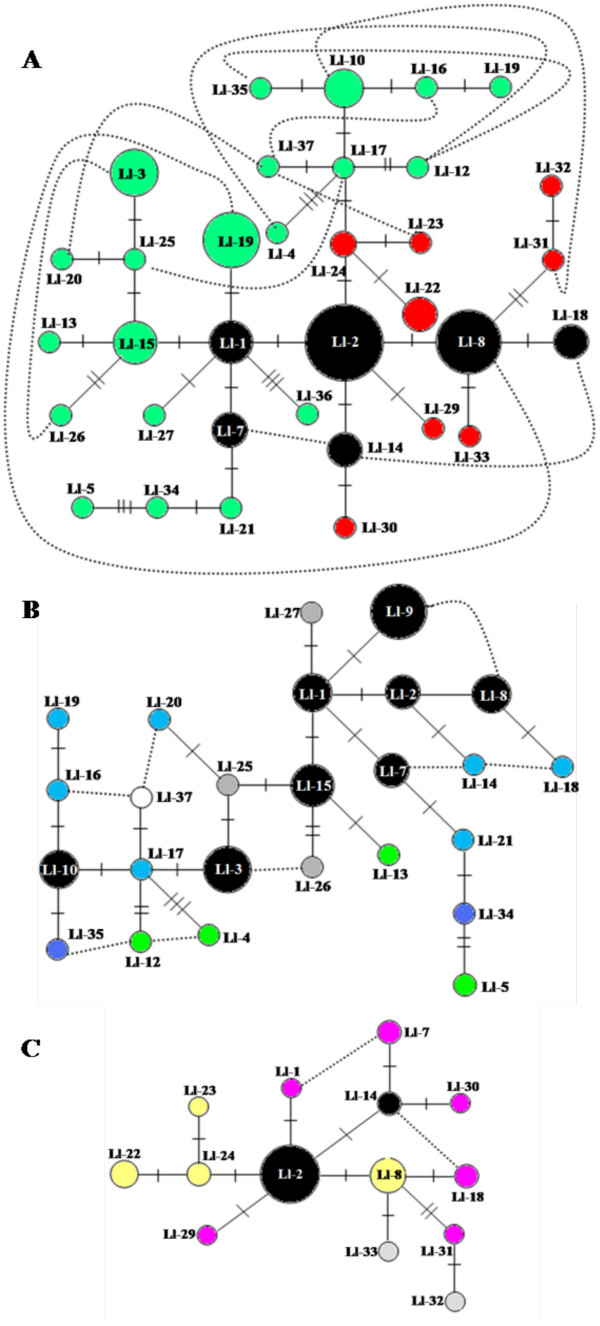
**Mantel Test**. Correlation between the genetic structuring and the geographical distances between the 9 populations of *Lu. longipalpis s.l*. used in this study.

To assess gene flow between the various populations investigated, we applied the minimum spanning network (MSN) analysis (Figure 4). The MSN constructed with all 34 different haplotypes (Figure 4A) shows that most of the haplotypes shared by groups <10°S and >10°S were positioned at the interior nodes, representing ancestral haplotypes [46,47]. Thus, the *Nm *of 2.22 migrates per generation between groups <10°S and >10°S may reflect a greater influence from the shared ancestral polymorphisms than by ongoing gene flow, according to the outline of the MSN. Nevertheless, ongoing gene flow between these populations is still possible, as alternate connections between the terminal haplotypes are observed (Figure 4A).

MSN analysis was separately applied to the population groups <10°S and >10°S (Figure 4B and 4C). In both cases, interior shared haplotypes were also observed for each group. However, the existence of shared haplotypes at the tips of the networks, and the connections between terminal haplotypes of distinct populations points to ongoing gene flow in each case, consistent with panmitic populations. For the group <10°S (Figure 4B) the strong gene flow is supported by the shared haplotype positioned at the tip (Ll-9), and by several connections between terminal haplotypes of distinct populations (Figure 4B). For the group >10°S, despite the presence of shared ancestral haplotypes, terminal haplotypes from three populations were connected (Figure 4C), suggesting ongoing gene flow.

Neutrality tests [48] performed between the groups <10°S and >10°S resulted in negative values. Although the estimates of *θs *and *θπ *are higher than those of *π*, the *D *test [31] and the D* and F* tests [32] were not significant (Tables 3 and 7). On the other hand, the *Fs *test [33] showed significant values for the same population groups. The mismatch distribution for groups <10°S and >10°S was unimodal (not shown), with very small raggedness statistics (0.025 and 0.050, respectively).

**Table 7:** Neutrality tests.

Groups	Tajima's *D*	Fu and Li's D*	Fu and Li's F*	Fu's F*s*
<10°S	-0.59541	-2.05938	-1.83948	-17.846^(1)^
>10°S	-1.17300	-0.96221	-1.20805	-8.309^(1)^

Neutrality tests (for both neutral evolution and population demography) were performed as indicated in the text. Significant values were those which P < 0.05, indicated by ^(1)^.

The genetic distances (*K2P*) between groups was 1.2%, between groups <10°S and >10°S. Based on *cyt b *molecular clock, the divergence time between these groups were estimated to be 0.45 million years ago (MY).

The model of isolation by distance was tested through linear regression between the geographic distances and the values of *F*_*ST*_/(1-*F*_*ST*_). We found that the pattern of genetic structuring of the populations was in disagreement with the model of isolation by distance (r = 0.347, P = 0.9628; Figure 3).

## Discussion

According to [14], there are five reproductively isolated populations of *L. longipalpis s.l*. in Brazil separated on the basis of chemotypes of the sexual pheromones they produce. The sexual pheromone 9MGB (chemotype 1) is produced by *Lu. longipalpis s.l *from Lapinha (MG); 3MαH (chemotype 2) is produced by flies from Jacobina (BA); cembrene-1 (chemotype 3) is found in flies from Sobral (CE), Santarem (PA), and Estrela de Alagoas and Costa do Sol (AL); cembrene-2 (chemotype 4) by flies from Jaiba (MG); finally, 9MGB+ (chemotype 5) by flies from Sobral (CE) and Montes Claros (MG).

Here, the genetic structuring of ten *Lu. longipalpis s.l*. populations from eastern Brazil was estimated through the analysis of 26 segregating sites of a partial segment of 261 nucleotides from the mitochondrial gene *cyt b*. The populations studied were clustered in two groups (as indicated in Materials and Methods), with individuals in group <10°S correspond to the sand flies that produce the chemotype 3 (cembrene-1 and 9MGB+); and those in the group >10°S to chemotypes 1, 2 and 5 (9MGB, 3MαH and 9MGB+).

Intra-population analyses of groups <10°S and >10°S revealed low and moderate genetic differentiation. However, in both cases, the values of *Nm *(Tables 4) were above the threshold required for differentiation by genetic drift (*Nm *< 2, [49]). Also, the presence of several tip connections between haplotypes of distinct populations, and a shared tip haplotype in the MSN for the group <10°S (Figure 4B) suggest that ongoing gene flow is likely responsible for the *Nm *value observed.

In contrast, inter-population analyses revealed moderate genetic differentiation and effective number of migrants between the groups <10°S and >10°S (Table 6). The *Fs *test and the smoothness of the mismatch distribution indicated that these groups are in geographic expansion. Since population expansion interferes with the estimates of gene flow [39-41], the *Nm *values obtained may be overestimated obscuring the actual population structuring. This, along with the prevalence of ancient introgression, rather than ongoing gene flow, indicates that genetic drift is mainly responsible for the moderate genetic differentiation between these groups.

Our results from the *cyt b *molecular clock suggest that the time of divergence between the *Lu. longipalpis s.l*. in groups <10°S and >10°S occurred around 0.45 MY. A similar divergence period (0.39 MY) was estimated between the populations of Baturite (located north the 10°S parallel) and Jacobina (located south of the same parallel). That estimation was based on the analysis of the molecular clock of the cytochrome oxidase II gene, according to a genetic distance of K2P = 0.009 [7], and a rate of divergence of 2.3% per MY [37]. The estimated time of divergence between the two groups investigated here correspond to the Mindel glaciation (0.38–0.41 MY) that took place during the Early to Middle Pleistocene, and was associated with the shift in the Sao Francisco River's course [16].

The Sao Francisco River is the second largest river in Brazil, separating the States of Pernambuco (PE) and Ceara (CE) from Bahia (BA) and Minas Gerais (MG). On its original course, the river flowed from MG, bearing northward through BA and continuing towards the coast of the State of Piaui (PI) through what is today the Piaui River and the Parnaiba River Basins. Following the uplift of the Parnaiba River Basin and the formation of a transversal geological fault to Northeastern coast, the course of the Sao Francisco River was altered to its current location [16], as shown in Figure 1A.

The reduced gene flow between sibling species and moderate genetic differentiation between groups <10°S and >10°S are consistent with a distribution pattern likely driven by the Sao Francisco River. Additionally, the estimated time of the divergence of these two groups coincides with the change of the river course. However, this divergence cannot be associated with reduced gene flow as mitochondrial DNA is prone to introgress through incipient species boundaries [50], and as demonstrated via *cyt b *gene analysis of *Lu. longipalpis s.l*. sympatric cryptic species [51]. Taken together, the data suggest that the Sao Francisco River is a significant geographic barrier between populations of *Lu. longipalpis s.l*., and could also have contributed to the current level of population diversity seen for this sand fly.

This putative role of the Sao Francisco River in the speciation of *Lu. longipalpis s.l*. may also explain the current distribution of male sexual pheromones chemotypes. A plausible scenario is based on the suggestion that 9MGB represents an ancestral chemotype pheromone and that the diterpenes (i.e., cembrene-1 and 2) are more recent chemotypes [14]. Dispersal of *Lu. longipalpis s.l*. belonging to the Brazilian clade [7] is estimated to have occurred in the Plioceno-Pleistocene [52] when the Sao Francisco River flowed on a South-to-North direction [16]. Thus, the Sao Francisco River would have served as a barrier to the introduction of the 9MGB chemotype in the Brazilian NE coast.

The current chemotype distribution in the Brazilian NE possibly followed a process similar to what is described for the ring species hypothesis [53]. Under this hypothesis *Lu. longipalpis s.l*. would have reached the coastal areas of the NE by circumventing the River's nascent, along its west margin, in Minas Gerais, South Eastern Brazil (as shown in Figure 1B). This process led to the adaptation to different habitats, and to differentiation into a population producing the cembrane-1 chemotype presently found in the NE of Brazil. This idea is supported by the presence of *Lu. longipalpis s.l*. in the Brazilian SE producing different pheromone chemotypes, such as 9MGB found in Lapinha Cave, cembrene-2 in found in Jaiba, and 3MαH found in Jacobina [14,54]. This scenario also supports the suggestion that *Lu. longipalpis s.l*. sibling species currently found in Sobral were the result of vicariance 1MY ago [55], and prior to the change in the course of the Sao Francisco River. Thus, the sympatric species in Sobral were isolated by the Sao Francisco River, leading to the development of 9MGB+ on the west and cembrene-1 on the east margins. Following the shift of the Sao Francisco River these species reached the current secondary contact proposed by [55] (Figure 1A). Our results suggesting greater similarity between *Lu. longipalpis s.l*. from Juazeiro (located on the southern rim of the Sao Francisco River) and flies belonging to group <10°S in comparison to flies belonging to group >10°S can be explained by a recent break-down of this geographic barrier, either by a recent geographic accident or through human activity in the region. This is consistent with our data indicating that no ongoing mitochondrial DNA haplotype exchange is detected between individuals from Juazeiro and individuals from group >10°S (Figure 4A).

*Lu. longipalpis s.l*. secreting cembrene-1 also are present in areas other than the Brazilian NE [14,54,56]. In two such areas (Marajo Island and Santarem), the presence of cembrene-1 was possibly due to a recent geographic expansion after the change in course of the Sao Francisco River. A study using microsatellite analysis indicated strong similarities between *Lu. longipalpis s.l*. from Marajo Island and flies from either Sobral or Natal [56]. Accordingly, our data supports a pattern compatible with population expansion from the latter two locations towards the former based on the mismatch distribution. In regards to the cembrene-1 population currently found in Espirito Santo do Pinhal, Sao Paulo [57], biogeographic and genetic studies are necessary in order to explain its origin.

## Conclusion

Based on the data presented here we propose that the Sao Francisco River restricted gene flow between *Lu. longipalpis s.l*., participating in speciation processes of this sand fly. This paper contributes to our understanding on the expansion of *Lu. longipalpis s.l*. in Brazil and provides novel clues in regards to several aspects of the divergence of this important vector species.

## Competing interests

The authors declare that they have no competing interests.

## Authors' contributions

IVCA participated in the conception of the study, analyses of the data, and drafting of the manuscript; IVS participated in data analyses and design of the study; JAF and MAM participated in sample collection; VQB and MRO participated in the conception and design of the study and crafting of the manuscript.
