# *Lutzomyia longipalpis s.l. *in Brazil and the impact of the Sao Francisco River in the speciation of this sand fly vector

**DOI:** 10.1186/1756-3305-1-37

**Published:** 2008-10-03

**Authors:** Iliano V Coutinho-Abreu, Ivan V Sonoda, Jose A Fonseca, Marcia A Melo, Valdir Q Balbino, Marcelo Ramalho-Ortigão

**Affiliations:** 1 Departamento de Genética, Centro de Ciências Biológicas, Universidade Federal de Pernambuco, Recife, Brazil; 2 Universidade Federal do Piauí, Teresina, Brazil; 3 Universidade Federal de Campina Grande, Campina Grande, Brazil; 4 Department of Biological Sciences, University of Notre Dame, Notre Dame, IN 46556, USA; 5 Department of Entomology, K-State University, 123 Waters Hall, Manhattan, KS 66506-4004, USA

## Abstract

In our recently published article "*Lutzomyia longipalpis s.l. *in Brazil and the impact of the Sao Francisco River in the speciation of this sand fly vector" by Iliano V. Coutinho-Abreu *et al*. a sentence located in paragraph 8 in the Discussion section had its meaning altered due to the improper insertion of three words.

## Correction

In our recently published article a sentence located in paragraph 8 in the Discussion section had its meaning altered due to the improper insertion of three words [1]. The paragraph published reads: "The reduced gene flow between sibling species and moderate genetic differentiation between groups < 10°S and > 10°S are consistent with a distribution pattern likely driven by the Sao Francisco River. Additionally, the estimated time of the divergence of these two groups coincides with the change of the river course. However, this divergence cannot be associated with reduced gene flow as mitochondrial DNA is prone to introgress through incipient species boundaries (reference 50 in the original article), and as demonstrated via *cyt b *gene analysis of *Lu. longipalpis s.l. *sympatric cryptic species (reference 51 in the original article). Taken together, the data suggest that the Sao Francisco River is a significant geographic barrier between populations of *Lu. longipalpis s.l.*, and could also have contributed to the current level of population diversity seen for this sand fly."

The paragraph should have read "The reduced gene flow and moderate genetic differentiation between groups < 10°S and > 10°S are consistent with a distribution pattern likely driven by the Sao Francisco River. Additionally, the estimated time of the divergence of these two groups coincides with the change of the river course. However, this divergence cannot be associated with reduced gene flow between sibling species as mitochondrial DNA is prone to introgress through incipient species boundaries (reference 50 in the original article), and as demonstrated via *cyt b *gene analysis of *Lu. longipalpis s.l. *sympatric cryptic species (reference 51 in the original article). Taken together, the data suggest that the Sao Francisco River is a significant geographic barrier between populations of *Lu. longipalpis s.l.*, and could also have contributed to the current level of population diversity seen for this sand fly."

Additionally, the Figure 3 legend (Mantel Test) was erroneously inserted into Figure 4 (Minimum Spanning Networks (MSN) and vice versa.

We apologize for any confusion these may have caused.
